# Serum high-sensitivity C-reactive protein and dementia in a community-dwelling Japanese older population (JPSC-AD)

**DOI:** 10.1038/s41598-024-57922-1

**Published:** 2024-03-28

**Authors:** Ayumi Tachibana, Jun-ichi Iga, Tomoki Ozaki, Taku Yoshida, Yuta Yoshino, Hideaki Shimizu, Takaaki Mori, Yoshihiko Furuta, Mao Shibata, Tomoyuki Ohara, Jun Hata, Yasuyuki Taki, Tatsuya Mikami, Tetsuya Maeda, Kenjiro Ono, Masaru Mimura, Kenji Nakashima, Minoru Takebayashi, Toshiharu Ninomiya, Shu-ichi Ueno, the JPSC-AD study group, Takanori Honda, Masato Akiyama, Shigeyuki Nakaji, Koichi Murashita, Kaori Sawada, Shintaro Yokoyama, Naoki Ishizuka, Hiroshi Akasaka, Yasuo Terayama, Hisashi Yonezawa, Junko Takahashi, Moeko Noguchi-Shinohara, Kazuo Iwasa, Sohshi Yuki-Nozaki, Masahito Yamada, Shogyoku Bun, Hidehito Niimura, Ryo Shikimoto, Hisashi Kida, Yasuyo Fukada, Hisanori Kowa, Toshiya Nakano, Kenji Wada, Masafumi Kishi, Tomohisa Ishikawa, Seiji Yuki, Ryuji Fukuhara, Asuka Koyama, Mamoru Hashimoto, Manabu Ikeda, Yoshihiro Kokubo, Kazuhiro Uchida, Midori Esaki, Yasuko Tatewaki, Benjamin Thyreau, Koji Yonemoto, Hisako Yoshida, Kaori Muto, Yusuke Inoue, Izen Ri, Yukihide Momozawa, Chikashi Terao, Michiaki Kubo, Yutaka Kiyohara

**Affiliations:** 1 Department of Neuropsychiatry, Neuroscience, Ehime University Graduate School of Medicine, Shitsukawa, Toon City, Ehime 791-0295 Japan; 2 Department of Neuropsychiatry, Matsukaze Hospital, Shikokuchuo, Ehime Japan; 3 Department of Psychiatry, Heisei Hospital, Ozu, Ehime Japan; 4 Department of Epidemiology and Public Health, Graduate School of Medical Sciences, Kyushu University, Fukuoka, Japan; 5 Department of Neuropsychiatry, Graduate School of Medical Sciences, Kyushu University, Fukuoka, Japan; 6 Department of Aging Research and Geriatric Medicine, Institute of Development, Aging and Cancer, Tohoku University, Sendai, Japan; 7 Department of Preemptive Medicine, Graduate School of Medicine, Hirosaki University, Hirosaki, Japan; 8 Division of Neurology and Gerontology, Department of Internal Medicine, School of Medicine, Iwate Medical University, Morioka, Iwate Japan; 9 Department of Neurology, Kanazawa University Graduate School of Medical Sciences, Kanazawa University, Kanazawa, Japan; 10 Center for Preventive Medicine, Keio University School of Medicine, Tokyo, Japan; 11 National Hospital Organization, Matsue Medical Center, Matsue, Shimane Japan; 12 Department of Neuropsychiatry, Faculty of Life Sciences, Kumamoto University, Kumamoto, Japan; 13 Ocular Pathology and Imaging Science, Kyushu University, Fukuoka, Japan; 14 Shonan Keiiku Hospital, Fujisawa, Japan; 15 Japanese Red Cross Morioka Hospital, Morioka, Japan; 16 Kitakami Saiseikai Hospital, Kitakami, Japan; 17 Kudanzaka Hospital, Tokyo, Japan; 18 Kawasaki Medical School, Kurashiki, Japan; 19 Tottori Red Cross Hospital, Tottori, Japan; 20 Kindai University Faculty of Medicine, Higashiosaka, Japan; 21 Osaka University Medical School, Suita, Japan; 22 National Cerebral and Cardiovascular Center, Suita, Japan; 23 Nakamura-Gakuen University, Fukuoka, Japan; 24 University of the Ryukyus, Nishihara, Japan; 25 Osaka Metropolitan University Graduate School of Medicine, Osaka, Japan; 26 University of Tokyo, Tokyo, Japan; 27 RIKEN Center for Integrative Medical Sciences, Yokohama, Japan; 28 Hisayama Research Institute for Lifestyle Diseases, Hisayama, Japan

**Keywords:** Alzheimer’s disease, Dementia, High-sensitivity C-reactive protein, Inflammation, Population-based study, Alzheimer's disease, Alzheimer's disease

## Abstract

In recent years, the association between neuroinflammatory markers and dementia, especially Alzheimer’s disease (AD), has attracted much attention. However, the evidence for the relationship between serum-hs-CRP and dementia including AD are inconsistent. Therefore, the relationships of serum high-sensitivity CRP (hs-CRP) with dementia including AD and with regions of interest of brain MRI were investigated. A total of 11,957 community residents aged 65 years or older were recruited in eight sites in Japan (JPSC-AD Study). After applying exclusion criteria, 10,085 participants who underwent blood tests and health-related examinations were analyzed. Then, serum hs-CRP levels were classified according to clinical cutoff values, and odds ratios for the presence of all-cause dementia and its subtypes were calculated for each serum hs-CRP level. In addition, the association between serum hs-CRP and brain volume regions of interest was also examined using analysis of covariance with data from 8614 individuals in the same cohort who underwent brain MRI. After multivariable adjustment, the odds ratios (ORs) for all-cause dementia were 1.04 (95% confidence interval [CI] 0.76–1.43), 1.68 (95%CI 1.08–2.61), and 1.51 (95%CI 1.08–2.11) for 1.0–1.9 mg/L, 2.0–2.9 mg/L, and ≥ 3.0 mg/L, respectively, compared to < 1.0 mg/L, and those for AD were 0.72 (95%CI 0.48–1.08), 1.76 (95%CI 1.08–2.89), and 1.61 (95%CI 1.11–2.35), for 1.0–1.9 mg/L, 2.0–2.9 mg/L, and ≥ 3.0 mg/L, respectively, compared to < 1.0 mg/L. Multivariable-adjusted ORs for all-cause dementia and for AD prevalence increased significantly with increasing serum hs-CRP levels (*p* for trend < 0.001 and *p* = 0.001, respectively). In addition, the multivariable-adjusted temporal cortex volume/estimated total intracranial volume ratio decreased significantly with increasing serum hs-CRP levels (< 1.0 mg/L 4.28%, 1.0–1.9 mg/L 4.27%, 2.0–2.9 mg/L 4.29%, ≥ 3.0 mg/L 4.21%; *p* for trend = 0.004). This study’s results suggest that elevated serum hs-CRP levels are associated with greater risk of presence of dementia, especially AD, and of temporal cortex atrophy in a community-dwelling Japanese older population.

## Introduction

Vascular inflammation and neuroinflammation have been implicated in increased risks of cognitive impairments, particularly Alzheimer’s disease (AD)^1–3^. Reports have been accumulating showing that systemic inflammation is directly associated with the development of AD^2,4,5^. Further, vascular risk factors such as hypertension, diabetes mellitus, and chronic kidney disease have been shown to be associated with AD^6–8^, and vascular inflammation is assumed to be behind these vascular risk factors^9^.

Serum high-sensitivity C reactive protein (hs-CRP) is a conveniently measured biomarker of systemic inflammation^10^, but there is no consensus on the association between serum hs-CRP and dementia including AD. Although serum hs-CRP has been reported to be significantly lower in AD patients than in healthy subjects^11^, a recent meta-analysis found that serum hs-CRP was significantly higher in AD patients than in healthy older subjects^12^. In another meta-analysis, serum hs-CRP was associated with all-cause dementia, but not with AD^13^. The relationship between serum hs-CRP and dementia has also been investigated in middle and old age groups, and many reports suggest that high serum hs-CRP levels in middle age increase the risk of AD^14^. However, reports on the relationship between serum hs-CRP and AD in older subjects were controversial^15,16^.

Regarding the association between serum hs-CRP levels and brain atrophy, there are reports that elevated serum hs-CRP levels were associated with atrophy of the frontal and temporal corticies^17^, and that serum hs-CRP was not significantly associated with brain atrophy after multivariable adjustment^18^. Thus, the results are inconsistent.

The Japan Prospective Studies Collaboration for Aging and Dementia (JPSC-AD) was a prospective cohort study of dementia. A total of 11,957 community-dwelling older residents were surveyed in eight sites from 2016 to 2018. The aim of this study was to examine the relationship between serum hs-CRP levels and dementia, especially AD, and their association with brain atrophy using cross-sectional data from this group of older Japanese persons.

## Methods

### Study population

**Figure 1:**
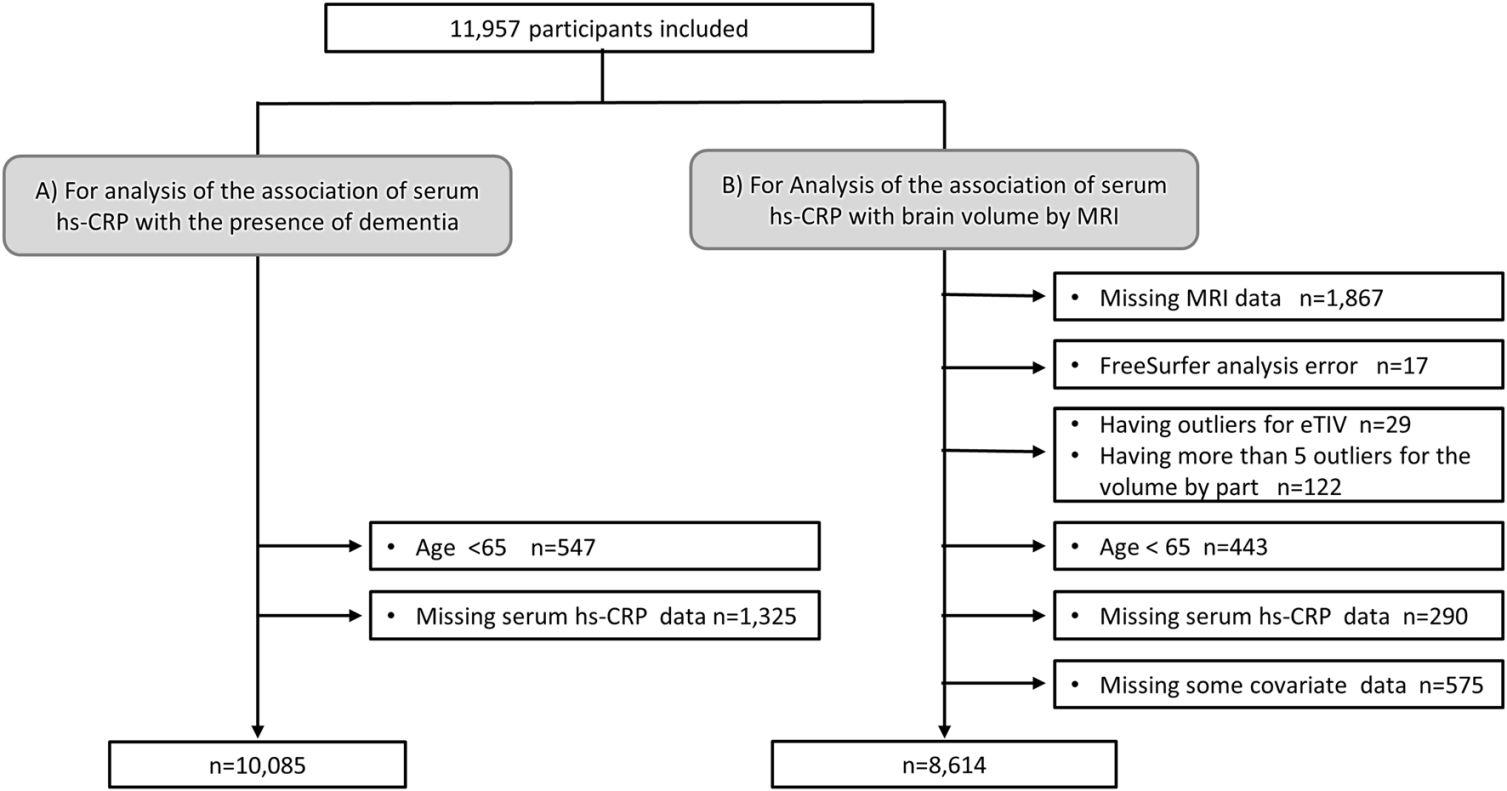
Flowchart of participants selection for the analysis of the association of serum high-sensitivity C-reactive protein with the presence of dementia (**A**) and brain volume estimated by brain magnetic resonance imaging (**B**). Abbreviations: MRI, magnetic resonance imaging; eTIV, estimated total intracranial volume.

The JPSC-AD Study was a multisite, population-based, prospective, cohort study of dementia involving eight sites in Japan with a pre-specified protocol and standardized measurement methods across the research sites^19–22^. The study was conducted in accordance with the Strengthening the Reporting of Observational Studies in Epidemiology (STROBE) checklist. The Medical Ethics Review Board of Ehime University (approval number 1610004) and Kyushu University Institutional Review Board for clinical research (approval number 686-10) approved this study. Written, informed consent was obtained from all participants. This study was performed in accordance with the Declaration of Helsinki. A total of 11,957 people consented to participate in the study between 2016 and 2018, of which 10,085 were eligible for the present study, excluding 547 participants younger than 65 years and 1325 participants lacking serum hs-CRP levels (Fig. 1A).

For the analysis of the association between serum hs-CRP levels and brain volume estimated by brain magnetic resonance imaging (MRI) scan, a total of 8614 participants were included after excluding 1867 participants without available brain MRI data, 17 participants for whom FreeSurfer analysis was errored, 29 participants who had outlier for estimated total intracranial volume (eTIV), 122 participants who had more than 5 outliers for the volume by part, 443 participants aged less than 65 years, 290 participants lacking serum hs-CRP levels, and 575 with missing some covariates data (Fig. 1B).

### Measurement of serum hs-CRP

Serum hs-CRP was measured at the central laboratory (LSI Medience Corporation, Tokyo) for all participants using the latex agglutination turbidimetry method. The detailed measurement methods of surum hs-CRP are shown in Supplemental Table 1. Basically, the samples were analyzed once and when abnormal values were found, reassay was carried out. Serum hs-CRP values were categorized into four categories (< 1.0 mg/L, 1.0–1.9 mg/L, 2.0–2.9 mg/L, ≥ 3.0 mg/L) according to clinical cut-off values.

**Table 1:** Clinical characteristics of the study participants according to serum high-sensitivity C-reactive protein level (n = 10,085).

Variable	Serum hs-CRP level, mg/L				
	< 1.0	1.0–1.9	2.0–2.9	≥ 3.0	*P* for trend
	n = 7465	n = 1379	n = 442	n = 799	
Age, y	73.5 (6.5)	74.0 (6.8)	74.2 (7.2)	75.4 (7.3)	< 0.001
Female, %	60.0	52.1	48.2	49.2	< 0.001
Education ≤ 9 y, %	31.4	31.6	33.6	37.2	0.002
Hypertension, %	72.0	78.6	77.9	78.0	< 0.001
Diabetes mellitus, %	15.2	19.82	22.5	21.5	< 0.001
Serum total cholesterol, mmol/L	5.4 (0.9)	5.3 (0.9)	5.2 (0.9)	5.0 (0.9)	< 0.001
BMI, kg/m^2^	23.0 (3.2)	24.4 (3.5)	24.3 (3.6)	23.9 (3.9)	< 0.001
Ischemic heart disease, %	4.2	5.2	5.4	7.0	< 0.001
Chronic kidney disease, %	17.4	23.8	24.7	26.7	< 0.001
ECG abnormalities, %	13.5	14.2	18.8	16.6	0.001
History of stroke, %	5.5	8.5	7.0	10.9	< 0.001
Current smoker, %	7.1	11.0	10.0	10.8	< 0.001
Current drinker, %	41.9	44.0	46.2	35.8	0.08
Regular exercise, %	43.3	38.0	35.4	36.9	< 0.001
APOE ε4 carrier, %	20.2	13.0	12.9	15.6	< 0.001
Depression, %	1.1	0.9	1.1	1.8	0.16

Values are shown as means (standard deviation) or frequencies.

*hs-CRP* high-sensitivity C-reactive protein, *BMI* body mass index, *ECG* electrocardiogram, *APOE* apolipoprotein E.

### Measurement of other risk factors

Each participant completed a self-administered questionnaire that included education, medical history, alcohol consumption, smoking, medical treatment (antihypertensive medications, diabetes medications), and physical activity. Blood pressure was measured three times with a sphygmomanometer at intervals of at least 5 min, and the average of the three measurements was used for the analysis^23^. Hypertension was defined by blood pressure greater than 140/90 mmHg or current use of antihypertensive medications. Ischemic heart disease was defined as history of myocardial infarction and/or coronary intervention. Diabetes mellitus was defined by a fasting glucose level ≥ 7.0 mmol/L, glucose level at any time ≥ 11.1 mmol/L, or HbA1c ≥ 6.5%, and/or current use of antidiabetic medications according to the 2010 American Diabetes Association (ADA) criteria^24^. Serum total cholesterol levels were measured by an enzymatic method. The body mass index (kg/m^2^) was calculated as an index of obesity. Chronic kidney disease was defined as estimated glomerular filtration rate < 60 ml/min/1.73 m^2^^25^ and calculated by multiplying the modified Chronic Kidney Disease Epidemiology Collaboration formula by the Japanese coefficient (0.813)^26,27^. Electrocardiographic abnormalities were defined as ST depression (Minnesota code, 4-1, 2, 3), left ventricular hypertrophy (3-1), and atrial fibrillation (8-3). Regular exercise was defined as physical activity performed for at least 30 min twice a week over the last year. Alcohol consumption and smoking were categorized as ongoing or not. Apolipoprotein E (APOE) ε4 carrier status was defined as the presence of the ε2/ε4, ε3/ε4, and ε4/ε4 alleles. To determine the APOE polymorphism, two single-nucleotide polymorphisms (rs429358 and rs7412) were genotyped using the multiplex PCR-based targeted sequencing method, as previously reported^28^. Depression was diagnosed according to the Diagnostic and Statistical Manual of Mental Disorders, Third Revised Edition^29^.

### Diagnosis of dementia

Dementia was diagnosed according to the Diagnostic and Statistical Manual of Mental Disorders, Third Revised Edition^29^. The diagnosis of AD was made based on the following criteria: the National Institute of Neurological and Communicative Disorders and Stroke and the Alzheimer’s Disease and Related Disorders Association criteria^30^. The diagnosis was made by the study team’s expert psychiatrists and neurologists using a standardized diagnostic system across the eight study sites. If the members of the endpoint determination committee agreed, the diagnosis was confirmed; if not, an endpoint determination committee was convened to confirm the diagnosis through discussion. The diagnostic procedure has been previously reported^22^. Dementia other than AD (isolated type and mixed type) was defined as non- Alzheimer’s dementia (non-AD).

### MRI analysis

Brain MRI equipment was set up according to the Alzheimer’s Disease Neuroimaging Initiative (ADNI) study brain MRI protocols^31^ and T1-weighted image (T1WI) parameters at all study sites. Details of the MRI at each site are provided in Supplemental Table 2.

**Table 2:** Age- and sex-adjusted and multivariable-adjusted odds ratios for the presence of all-cause dementia and dementia subtypes according to serum high-sensitivity C-reactive protein level.

Serum hs-CRP, mg/L	No. of cases/participants	Age- and sex-adjusted		Multivariable-adjusted^a^	
		OR (95%CI)	*p*	OR (95%CI)	*p*
All-cause dementia					
< 1.0	367/7465	1.00 (reference)		1.00 (reference)	
1.0–1.9	82/1379	1.08 (0.82–1.41)	0.60	1.04 (0.76–1.43)	0.80
2.0–2.9	38/442	1.57 (1.06–2.32)	0.03	1.68 (1.08–2.61)	0.02
≥ 3.0	86/799	1.70 (1.28–2.25)	< 0.001	1.51 (1.08–2.11)	0.02
*P* for trend		< 0.001		< 0.001	
Alzheimer’s disease (isolated type)					
< 1.0	269/7465	1.00 (reference)		1.00 (reference)	
1.0–1.9	46/1379	0.77 (0.55–1.09)	0.14	0.72 (0.48–1.08)	0.11
2.0–2.9	25/442	1.33 (0.84–2.11)	0.23	1.76 (1.08–2.89)	0.02
≥ 3.0	58/799	1.47 (1.06–2.04)	0.02	1.61 (1.11–2.35)	0.01
*P* for trend		0.004		0.001	
Non-Alzheimer’s dementia					
< 1.0	75/7465	1.00 (reference)		1.00 (reference)	
1.0–1.9	28/1379	1.84 (1.18–2.88)	0.007	2.02 (1.19–3.44)	0.009
2.0–2.9	7/442	1.32 (0.60–2.91)	0.50	0.64 (0.19–2.17)	0.48
≥ 3.0	20/799	1.80 (1.08–3.01)	0.03	1.06 (0.53–2.14)	0.87
*P* for trend		0.002		0.47	

Mixed types including Alzheimer’s disease are not included in either Alzheimer’s disease (isolated) or non-Alzheimer’s dementia groups.

*hs-CRP* high-sensitivity C-reactive protein, *OR* odds ratio, *CI* confidence interval.

^a^Adjusted for age, sex, low education, hypertension, ischemic heart disease, diabetes mellitus, serum total cholesterol, body mass index, chronic kidney disease, electrocardiogram abnormalities, history of stroke, smoking habit, alcohol intake, regular exercise, apolipoprotein E ε4 carrier status, depression, and research site.

Segmentation and volumetric measurements of cortical and subcortical brain structures were performed automatically using FreeSurfer software (http://surfer.nmr.mgh.harvard.edu), version 5.3. Total brain volume was calculated from brain segment volumes excluding ventricles. Cortical segmentation was performed using the Desikan-Killiany atlas^32^. The eTIV of each subject was also calculated using the standard FreeSurfer processing pipeline by exploiting the relationship between the intracranial volume and the linear transformation to the atlas template^33^. Specifically, FreeSurfer estimates of each brain parameter were fitted with a linear regression model adjusted for age, age squared, and sex. If the residuals of an individual participant’s data were less than − 4 standard deviation (SD) units or greater than + 4 SD units from the linear regression, these data were considered extreme outliers. Extreme outliers in eTIV and volumes of at least five brain regions were excluded.

We selected from the gray matter 4 representative cerebral lobes, i.e., the frontal cortex, parietal cortex, temporal cortex, and occipital cortex, and 3 dementia-related brain regions, i.e., the insular cortex, hippocampus, and amygdala, based on the findings of a previous study^34^.

### Statistical analyses

For patient background, comparisons among the four groups of serum hs-CRP < 1.0 mg/L, 1.0–1.9 mg/L, 2.0–2.9 mg/L, ≥ 3.0 mg/L were evaluated with the logistic regression analysis for binary variables and linear regression analysis for continuous variables. Age- and sex-adjusted prevalence of all-cause dementia and its subtypes was estimated by the direct method with 5-year age groups. Age- and sex-adjusted and multivariable-adjusted odds ratios for presence of all-cause dementia and its subtypes were calculated by logistic regression analysis. Trends between serum hs-CRP levels were calculated using multiple regression analysis. Multivariable adjustment was performed for age, sex, education, hypertension, ischemic heart disease, diabetes mellitus, serum total cholesterol level, BMI, chronic kidney disease, abnormal electrocardiogram (ECG), previous stroke, smoking, alcohol consumption, exercise habits, APOE ε4 carrier status, depression, and research site. Of the participants selected for this analysis, 7.7% (= 774/10,085) were excluded from the multivariable-adjusted analysis due to missing data for any covariates. Mean values of brain volume were adjusted for the same adjustment factors and subjected to analysis of covariance. Two-tailed *p* value < 0.05 were considered significant. In the analysis for the association between serum hs-CRP levels and each regional brain volume, false discovery rate (FDR)-corrected *p* values (i.e., q values) was calculated by using the Benjamini–Hochberg methods^35^ in consideration of multiple comparisons, and a q-value < 0.05 was considered statistically significant. All analyses were conducted using the Statistical Package for the Social Sciences (version 23; SPSS Inc., Chicago, IL, USA).

## Results

### Baseline characteristics of the study population

The patient background of the study population is summarized in Table 1. Age, low education, current smoker, BMI, hypertension, diabetes mellitus, ischemic heart disease, chronic kidney disease, stroke, and ECG abnormalities were significantly higher with higher serum hs-CRP levels. Frequency of female, serum total cholesterol levels, regular exercise, and frequency of APOE ε4 carriers were significantly lower with higher serum hs-CRP levels.

### Associations of serum hs-CRP levels with all-cause dementia and dementia subtypes

**Figure 2:**
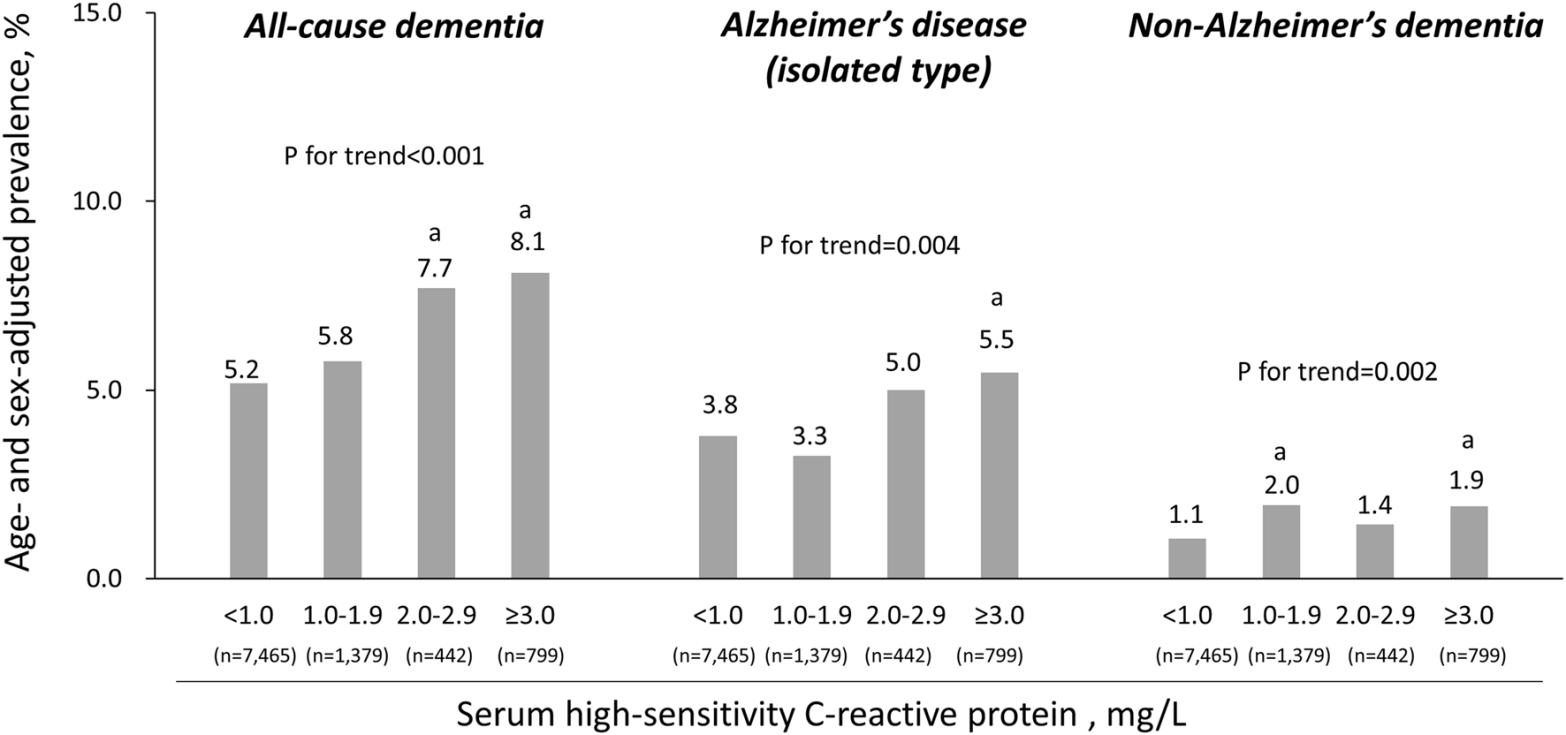
The age- and sex-adjusted prevalence of all-cause dementia and dementia subtypes according to serum high-sensitivity C-reactive protein level. The data are expressed as the prevalence after adjusting for age and sex. (a) *p* < 0.05, compared to the lowest serum high-sensitivity C-reactive protein group.

First, the age- and sex-adjusted prevalence of all-cause dementia, AD and non-AD are shown in Fig. 2. The age- and sex-adjusted prevalence of all-cause dementia, AD and non-AD increased significantly with rising serum hs-CRP levels (*p* for trend < 0.001, *p* for trend = 0.004, *p* for trend = 0.002, respectively). Moreover, the odds ratios of the presence of all-cause dementia, AD and non-AD according to serum hs-CRP level were calculated; as shown in Table 2. The multivariable-adjusted odds ratios for the presence of all-cause dementia were 1.04 (95% confidence interval [CI] 0.76–1.43), 1.68 (95%CI 1.08–2.61), and 1.51 (95%CI 1.08–2.11) for 1.0–1.9 mg/L, 2.0–2.9 mg/L, and ≥ 3.0 mg/L, respectively, compared to < 1.0 mg/L, with a significant increase with higher serum hs-CRP levels (*p* for trend < 0.001). Similarly, as shown in Table 2, the multivariable-adjusted odds ratios for the presence of AD were 0.72 (95%CI 0.48–1.08), 1.76 (95%CI 1.08–2.89), and 1.61 (95%CI 1.11–2.35), for 1.0–1.9 mg/L, 2.0–2.9 mg/L, and ≥ 3.0 mg/L, respectively, compared to < 1.0 mg/L, increasing significantly with higher serum hs-CRP levels (*p* for trend = 0.001). On the other hand, there was no evidence of significant associations between serum hs-CRP levels and the multivariable-adjusted odds ratio of non-AD (*p* for trend = 0.47). In a sensitivity analysis excluding 491 patients with serum hs-CRP ≥ 5.0 mg/L, the significant association between serum hs-CRP and not only all-cause dementia, but also AD, remained (Supplemental Table 3).

**Table 3:** Multivariable-adjusted mean values (95% confidence intervals) of total brain volume and each regional brain volume to estimated total intracranial volume ratios according to serum high-sensitivity C-reactive protein level in all participants.

Brain region	Serum hs-CRP levels					
	< 1.0 mg/L (n = 6412)	1.0–1.9 mg/L (n = 1178)	2.0–2.9 mg/L (n = 387)	≥ 3.0 mg/L (n = 637)	*P* for trend	q-value of FDR correction
Total brain volume/eTIV	54.59 (53.95 to 55.24)	54.51 (53.85 to 55.17)	54.56 (53.84 to 55.27)	54.24 (53.56 to 54.91)	0.02	–
Each regional brain^a^						
Frontal cortex volume/eTIV	6.85 (6.73 to 6.98)	6.86 (6.73 to 6.99)	6.84 (6.70 to 6.99)	6.79 (6.66 to 6.93)	0.051	0.18
Parietal cortex volume/eTIV	4.34 (4.24 to 4.43)	4.34 (4.24 to 4.44)	4.33 (4.22 to 4.43)	4.30 (4.20 to 4.49)	0.13	0.23
Temporal cortex volume/eTIV	4.28 (4.19 to 4.37)	4.27 (4.18 to 4.37)	4.29 (4.19 to 4.39)	4.21 (4.12 to 4.31)	0.004	0.03
Occipital cortex volume/eTIV	1.73 (1.68 to 1.77)	1.73 (1.68 to 1.78)	1.74 (1.69 to 1.79)	1.72 (1.67 to 1.76)	0.56	0.78
Insular cortex volume/eTIV	0.75 (0.73 to 0.76)	0.75 (0.73 to 0.76)	0.76 (0.74 to 0.77)	0.75 (0.73 to 0.76)	0.78	0.91
Hippocampal volume/eTIV	0.31 (0.30 to 0.32)	0.31 (0.30 to 0.32)	0.31 (0.30 to 0.33)	0.31 (0.30 to 0.32)	0.08	0.19
Amygdala volume/eTIV	0.018 (0.013 to 0.023)	0.018 (0.013 to 0.024)	0.019 (0.013 to 0.025)	0.018 (0.012 to 0.023)	0.91	0.91

Values are shown as multivariable-adjusted mean values (95% confidence intervals), where values are calculated as follows: ([left + right] volumes of total brain or each regional brain/eTIV) × 100 (%), where values were adjusted for the following covariates: age, sex, low education, hypertension, ischemic heart disease, diabetes mellitus, serum total cholesterol, body mass index, chronic kidney disease, electrocardiogram abnormalities, history of stroke, smoking habit, alcohol intake, regular exercise, apolipoprotein E ε4 carrier status, depression, and research site.

*hs-CRP* high-sensitivity C-reactive protein, *eTIV* estimated total intracranial volume, *FDR* false discovery rate.

^a^The number of missing data for each regional brain volume are 93 for fontal cortex, 20 for parietal cortex, 81 for temporal cortex, 33 occipital cortex, 10 for insular cortex, 11 for hippocampus, and 11 for amygdala.

### Associations of serum hs-CRP levels with brain volume

Next, Table 3 shows the relationships between serum hs-CRP levels and each brain volume/total intracranial volume ratio. In all subjects included in the imaging analysis, the multivariable-adjusted mean value of total brain volume/eTIV ratio decreased significantly with increasing serum hs-CRP levels (*p* for trend = 0.02) (Table 3). With regard to regional brain volume, higher serum hs-CRP levels were associated significantly with lower multivariable-adjusted mean values of temporal cortex volume/eTIV ratio (*p* for trend = 0.004). This significant association was also observed, even after FDR correction (q-value = 0.03). Sensitivity analysis after excluding 353 participants with dementia found a similar negative association between serum hs-CRP levels and mean values of temporal cortex volume/eTIV ratio (%): serum hs-CRP < 1.0 mg/L, 4.33 (95%CI 4.24–4.42); 1.0–1.9 mg/L, 4.33 (4.24–4.42); 2.0–2.9 mg/L, 4.36 (4.26–4.46); ≥ 3.0 mg/L, 4.27 (4.18–4.37); *p* for trend = 0.02 (data not shown).

## Discussion

In this large cohort study of Japanese older people, the odds ratios for the presence of all-cause dementia and of AD were positively correlated with serum hs-CRP levels. In addition, the temporal cortex volume/eTIV ratio was inversely correlated with serum hs-CRP levels. This significant relationship also was maintained in a sensitivity analysis after excluding patients with dementia.

Though recent reports that vascular inflammation and neuroinflammation are risk factors for AD have accumulated^1–3^, reports on the association between serum hs-CRP levels and dementia including AD are inconsistent. A recent meta-analysis found that serum hs-CRP was associated with all-cause dementia, but not with AD^13^. In contrast, the present study showed that elevated levels of serum hs-CRP contributed to an increased odds ratio not only for all-cause dementia, but also for AD alone, and furthermore did not contribute to a significant increased odds ratio for non-AD. The multivariable-adjusted odds ratios for the presence of all-cause dementia and AD for every 1 SD increment in log (serum hs-CRP) were 1.06 (95% confidence interval [CI] 0.97–1.15) and 1.05 (95%CI 0.95–1.15), respectively, which were not significant. The results show that there may not be a linear relationship between hs-CRP and dementia and between hs-CRP and AD, but there is a relationship between elevated hs-CRP and dementia, especially AD. Regarding the association between serum hs-CRP and brain atrophy, previous studies have reported that elevated serum hs-CRP is associated with temporal lobe atrophy^17,35^, and the present results were consistent with those findings. A longitudinal study using data from the Framingham Offspring Study found no significant association between elevated serum hs-CRP levels and brain atrophy after multivariable adjustment (adjustment factors: age, sex, time to MRI scan, educational history, and APOE ε4 carrier status), and elevated serum hs-CRP levels were significantly associated with temporal lobe atrophy in APOE ε4 carriers^18^. The present study had a larger sample size than these existing studies, and it included potential confounders not addressed in other studies as adjustment factors.

It is widely known that an inflammatory response occurs in the brains of AD patients. Activation of microglia, immunocompetent cells that release inflammatory cytokines, has been reported to occur around the senile plaque in AD brains^36^. The association of peripherally derived serum hs-CRP with AD may be explained by the fact that peripheral inflammation transfers into the brain via disruption of the blood–brain barrier^37,38^. Recent studies have also shown that the migration of immune cells of peripheral origin, such as T cells and neutrophils, into the brain is involved in the brain aging and neurodegeneration^39–41^. Interestingly, serum hs-CRP itself was reported to increase the permeability of the cerebral blood–brain barrier^42^. In addition, an association between cerebral small vessel disease (white matter lesions, perivascular space, etc.) and risk of dementia, especially AD, has recently been reported^43–46^, and vascular risk factors are associated with the development of cerebral small vessel disease^47^. The background of the involvement of vascular risk factors in AD can be related to vascular inflammation, i.e., inflammatory markers.

The fact that elevated levels of inflammation were associated with temporal cortex atrophy in the present study suggests that the temporal cortex is particularly vulnerable to inflammation among brain regions. Differences in the distribution pattern of inflammatory cytokine receptors may underlie why some brain regions are more vulnerable to inflammation. In AD, the density and expression of inflammatory cytokine receptors has been reported to be increased in neurodegenerative regions, including the prefrontal cortex and medial temporal lobes^48^. Interestingly, plasma inflammation predicted phenotypic conversion and clinical progression of autosomal dominant frontotemporal lobar degeneration^49^. It has also been reported that higher serum hs-CRP levels were associated with decreased frontotemporal functional network connectivity in community-dwelling older adults^50^.

The strengths of this study are the large sample size and the ability to adjust for potential confounding factors. However, there are some limitations. First, because this was a cross-sectional study, the causal relationships among serum hs-CRP, dementia including AD, and brain volume cannot be addressed. In addition, the ability to generalize the results of this study to other racial groups is limited due to differences in lifestyle and background. Longitudinal, world-wide, and basic medical studies are needed to clarify the mechanism of the association between inflammation and AD.

## Conclusions

This study’s results suggest that elevated serum hs-CRP levels are associated with greater risk of presence of dementia, especially AD, and of temporal cortex atrophy in a community-dwelling Japanese older population.

### Supplementary Information

Supplementary Tables.

## Data Availability

All the processed data generated during this study are provided in the main article and Supplementary Information. The raw data are not openly available to protect the confidentiality of participants and to comply with the terms of participant consent. Requests related to the raw data should be addressed to the principal investigator, Toshiharu Ninomiya (Department of Epidemiology and Public Health, Graduate School of Medical Sciences, Kyushu University, Fukuoka, Japan) [t.ninomiya.a47@m.kyushu-u.ac.jp], and the Japan Agency for Medical Research and Development.
