# Supplementary material for: Serum high-sensitivity C-reactive protein and dementia in a community-dwelling Japanese older population (JPSC-AD)
Source: Sci Rep. 2024 Mar 28;14:7374. doi: 10.1038/s41598-024-57922-1 (PMC10978957; doi:10.1038/s41598-024-57922-1)
Supplement: Supplementary file 1 — Supplementary Tables. [file 41598_2024_57922_MOESM1_ESM.pdf]

Supplemental Table 1: Methods of serum hs-CRP measurement

Reagent	Reagent name	N-assay LA CRP-U
	Product number	07E1X80001015330
	Manufacturing company	Nittobo Medical, Tokyo, Japan
Measuring instrument	Measuring instrument	JCA-BM9130
	Manufacturing company	Fukuda Denshi, Tokyo, Japan
	Measuring method	Latex agglutination turbidimetry method
Standardization	Reference material for calibration	IRMM ERM-DA470

Supplemental Table 2: MRI instruments and acquisition

Research site	Measuring instrument	Manufacturer	Field strength	Sequence	TR/TE/TI (ms)	Flip angle (°)	FOV (mm)	Acquisition matrix	Voxel size (mm)
Hirosaki City, Aomori	Signa HDxt	GE Healthcare	3 tesla	SPGR	10/4.1/700	10	260	256 × 256	1.0 × 1.0 × 1.2
Yahaba Town, Iwate	Intera	Philips	1.5 tesla	MPRAGE	8.6/4.0/1000	8	240	192 × 192	0.94 × 0.94 × 1.2
Nakajima Town, Nanao City, Kanazawa	ECHELON	Hitachi	1.5 tesla	MPRAGE	9.2/4.0/1034	8	240	192 × 192	0.94 × 0.94 × 1.2
Arakawa Ward, Tokyo	MAGNETOM Spectra	Siemens	3 tesla	MPRAGE	2300/3.0/900	9	270	240 × 240	1.1 × 1.1 × 1.2
Ama Town, Shimane	Achieva	Philips	1.5 tesla	MPRAGE	8.6/4.0/1000	8	240	240×240	0.94×0.94×1.2
Nakayama Town, Iyo City, Ehime	Achieva	Philips	1.5 tesla	MPRAGE	8.6/4.0/1000	8	240	192 × 192	0.94 × 0.94 × 1.2
Hisayama Town, Fukuoka	Intera	Philips	1.5 tesla	MPRAGE	8.5/4.0/1000	8	240	192 × 192	0.94 × 0.94 × 1.2
Arao City, Kumamoto	Ingenia<br>Signa HDxt	Philips<br>GE Healthcare	1.5 tesla<br>1.5 tesla	MPRAGE	8.6/4.0/1000<br>8.3/3.3/1000	9<br>8	240	192 × 192	0.94 × 0.94 × 1.2

Abbreviations: TR, repetition time; TE, echo time; TI, inversion time; FOV, field of view; GE, General Electric Company; SPGR, spoiled gradient recalled echo; MPRAGE, magnetization prepared rapid acquisition gradient echo.

Supplemental Table 3. Age- and sex-adjusted and multivariable-adjusted odds ratios for the presence of all-cause dementia and dementia subtypes according to serum high-sensitivity C-reactive protein level after excluding participants with serum hs-CRP of ≥5.0 mg/L

Serum hs-CRP, mg/L	No. of cases/ participants	Age- and sex-adjusted OR (95%CI)	Age- and sex-adjusted p	Multivariable-adjusted [a)] OR (95%CI)	Multivariable-adjusted [a)] p
All-cause dementia					
<1.0	367/7465	1.00(reference)		1.00(reference)	
1.0-1.9	82/1379	1.08(0.82-1.41)	0.60	1.04(0.76-1.44)	0.78
2.0-2.9	38/442	1.57(1.06-2.32)	0.03	1.69(1.09-2.64)	0.02
≥3.0	36/308	1.80(1.18-2.75)	0.007	1.84(1.12-3.00)	0.02
P for trend		<0.001		<0.001	
Alzheimer's disease (isolated type)					
<1.0	269/7465	1.00(reference)		1.00(reference)	
1.0-1.9	46/1379	0.77(0.55-1.09)	0.14	0.72(0.48-1.09)	0.12
2.0-2.9	25/442	1.33(0.83-2.11)	0.24	1.78(1.09-2.92)	0.02
≥3.0	23/308	1.40(0.85-2.30)	0.19	1.81(1.04-3.14)	0.04
P for trend		0.046		0.001	
Non-Alzheimer's dementia					
<1.0	75/7465	1.00 (reference)		1.00 (reference)	
1.0-1.9	28/1379	1.84 (1.18-2.87)	0.007	2.02 (1.19-3.43)	0.01
2.0-2.9	7/442	1.32 (0.60-2.91)	0.50	0.64 (0.19-2.17)	0.48
≥3.0	12/308	2.80 (1.47-5.34)	0.002	1.80 (0.70-4.59)	0.22
P for trend		<0.001		0.08	

Abbreviations: hs-CRP, high-sensitivity C-reactive protein; OR, odds ratio; CI, confidence interval.

a) Adjusted for age, sex, low education, hypertension, ischemic heart disease, diabetes mellitus, serum total cholesterol, body mass index, chronic kidney disease, electrocardiogram abnormalities, history of stroke, smoking habit, alcohol intake, regular exercise, apolipoprotein E ε4 carrier status, depression, and research site.
